# Knowledge for effective action to improve the health of women, children and adolescents in the sustainable development era

**DOI:** 10.2471/BLT.16.174243

**Published:** 2016-05-02

**Authors:** Flavia Bustreo, Robin Gorna, David Nabarro

**Affiliations:** a Family, Women’s and Children’s Health, World Health Organization avenue Appia 20, 1211 Geneva 27, Switzerland.; b Partnership for Maternal, Newborn & Child Health, World Health Organization, Geneva, Switzerland.; c United Nations, New York, United States of America.

January 2016 marked the beginning of a new era for health and development. It was the start of *Transforming our world: the 2030 agenda for sustainable development* and its accompanying sustainable development goals (SDGs).^1^ All governments have committed to this ambitious sustainable agenda and its goals. 

The *Global strategy for women’s, children’s and adolescents’ health (2016–2030)*^2^ and its operational framework are aligned with the SDGs and provide an evidence-based roadmap for ending preventable deaths of women, children and adolescents by 2030. The global strategy can guide collective action so that every woman, child and adolescent – even those living in the most challenging settings – can achieve their full potential and rights to health and well-being.

Governments are aligning their work with the SDGs and the global strategy’s three objectives – survive, thrive and transform – in ways that meet their countries’ priorities and unique contexts. The survival, health and well-being of women, children and adolescents are essential to achieving the SDGs. Analysis of lessons from the millennium development goal (MDG) implementation process – what has worked and what hasn’t – is needed to effectively implement the agenda set by the SDGs and the global strategy.

Achieving the global strategy and the SDGs will require the use of the best available knowledge for action, as well as investment in new research and innovation. This month’s *Bulletin* theme issue seeks to broaden the evidence on effective country implementation and lessons learnt from the MDGs. Kuruvilla et al.^3^ summarize the current global strategy, show how the objectives of the strategy are aligned with the SDGs and how selected countries are already making progress.

Several papers in this issue deal with progress on the survival objective of the global strategy; Negandhi et al.^4^ present a surveillance-based maternal and infant death review system in India; Murguía-Peniche et al.^5^ and McKinnon et al.^6^ address under-researched topics, such as the factors associated with stillbirths in Mexico and the high prevalence of suicidal behaviours among adolescents in low- and middle-income countries, respectively.

The strategy’s thrive objective addresses the overall health and well-being of mothers, children and adolescents. Chai et al.^7^ determine how exposure to violence hinders child development and can affect health across the life-course and subsequent generations. Askew et al.^8^ describe the importance of ensuring sexual and reproductive health and rights in humanitarian settings.

The transform objective of the strategy focuses on expanding enabling environments and aims to transform societies so that women, children and adolescents everywhere can realize their rights to the highest attainable standards of health and well-being. Several papers address the global strategy’s transform objective. Newberry et al.^9^ present a formal emergency response infrastructure developed in India for gender-based violence.

This special issue also includes papers on approaches that have helped countries achieve improved health outcomes for women, children and adolescents. Marston et al.^10^ discuss the importance of community engagement in achieving results. Ahmed et al.^11^ describe policies and programmes that contributed to reductions in child and maternal mortality. Frost et al.^12^ explain how multistakeholder dialogues are used to clarify what works and does not work in policy-making and implementation.

To drive the global strategy forward, governments, civil society, the private sector and other partners have mobilized under the banner of Every Woman Every Child and a group of political champions has been formed. A global financing facility has been established as a country-led partnership that combines domestic, external, and innovative financing for health and development. An independent accountability panel has been appointed by the United Nations Secretary-General to report on results, resources and rights for women’s children’s and adolescents’ health. This month, the World Health Assembly will consider a resolution on implementing the global strategy, giving delegations an opportunity to discuss its content and shape their own commitments. In addition, this theme issue illustrates the importance of continued investments in research and learning to support country implementation.

The generation and sharing of knowledge for action is essential to achieve the global strategy’s objectives, to secure a healthy and transformative future for women, children and adolescents, their families and communities in the SDG era.
